# Evidence Based Analysis Enhances Surgical Outcomes of Novice Resident Surgeons

**DOI:** 10.3390/vision9030052

**Published:** 2025-07-03

**Authors:** Neel K. Patel, Kenneth L. Cohen

**Affiliations:** 1 Department of Ophthalmology, School of Medicine, The University of North Carolina at Chapel Hill, 5151 Bioinformatics Bldg., CB# 7040, Chapel Hill, NC 27599, USA; n_patel1023@email.campbell.edu; 2 The Kittner Eye Center, 2226 Nelson Highway, Suite 200, Chapel Hill, NC 27517, USA; 3 School of Osteopathic Medicine, Campbell University, P.O. Box 4280, Buies Creek, NC 27506, USA

**Keywords:** resident cataract surgery, quality improvement, evidence based analysis, refractive outcomes, optimization, effective lens position

## Abstract

Evidence based practice enhances healthcare delivery and prevents unsafe procedures. While competency based assessments of resident cataract surgery are standard, evidence based analysis of refractive outcomes remains underutilized in educational curricula. This retrospective single center study evaluated refractive outcomes from 21 novice ophthalmology resident surgeons. Three independent groups were compared based on formal constant optimization for intraocular lens (IOL) calculation: non-optimized Haigis (n = 216), a0-optimized (n = 94), and a0/a1/a2-optimized (n = 121). All surgeries were supervised by a single attending surgeon. Mean absolute error (MAE) and the percentage of eyes within ±0.25 D and ±0.50 D of predicted spherical equivalent (SEQ) were calculated. Also, systematic bias in effective lens position (ELP) was analyzed to update manufacturer IOL constants. MAE improved from 0.44 D (non-optimized) to 0.35 D (a0-optimized *p* = 0.009) and 0.19 D (a0/a1/a2-optimized *p* < 0.001). The percentage within ±0.50 D increased from 65.7% to 74.4% to 95.0%, respectively. With ELP bias correction, updated A constant and ACD were 119.266 and 5.755 mm. a0/a1/a2-optimized outcomes were comparable to ELP bias correction for the Barrett UII, Kane, and Hill-RBF formulas. Evidence based optimization of IOL constants significantly enhances novice resident surgical outcomes, achieving parity with prediction models. A formal curriculum on IOL calculation and optimization is warranted.

## 1. Introduction

Ophthalmology residency programs are faced with the challenge of defining and measuring surgical competency [1,2]. To this end, competency based curricula have been developed. Standardized, objective competency based evaluation of surgical skills is well established using defined tasks and skills assessment real-time in the operating room and wet-labs [3,4,5].

Evidence based practices are clinical decisions based on using the best available clinical data. For quality improvement of patient care and to reduce variability of patient outcome, evidence based practice is necessary. It is recommended that educational curricula train health care students in evidence based practice [6]. However, to our knowledge, evidence based data used for quality improvement of patient outcomes are missing from the evolution of the formalized teaching of modern cataract surgery [7]. Evidence based optimization of intraocular lens (IOL) constants is essential for quality improvement of patient refractive outcomes [8,9,10,11]. Therefore, the advantages and main objective of our study are to educate ophthalmology residents in evidence based practice, which should improve the quality of health care. Specifically, our study uses available data to perform evidence based optimization of IOL calculation formula constants and minimize systematic bias of the effective lens position (ELP) [8,9,12]. Importantly, the evidence based results are used proactively, and these results are evaluated.

## 2. Materials and Methods

### 2.1. Subjects

This is a retrospective study of 431 patients, who agreed to undergo routine cataract surgery performed by 21 different novice resident surgeons during their first or second year in the ophthalmology residency program at the University of North Carolina at Chapel Hill, School of Medicine, Department of Ophthalmology from May 2017 through August 2023. Senior residents were not included. Inclusion criteria were age greater than 18 years, a clinically significant cataract, and agreeing to have an ophthalmology resident as primary surgeon. Postoperative measurements were at approximately one month. Patients with previous ocular surgery, intraoperative and postoperative complications and comorbidities that would likely affect visual acuity outcome were excluded. The first ten cases for each resident were excluded to act as a buffer for novice surgeons to become acclimated to routine cataract surgery. For bilateral surgeries, only the first operated eye was included to support random laterality.

The University of North Carolina Office of Human Research and Ethics issued a waiver of informed consent in its entirety as well as a waiver of HIPAA authorization. This study was in adherence to the tenets of the Declaration of Helsinki as well as regulations established by HIPAA.

For the first 167 patients, biometry was performed using partial coherence interferometry (PCI) with the IOLMaster 500 (Carl Zeiss Meditec AG, Dublin, CA, USA). For the subsequent 264 patients, biometry was performed using SWEPT Source OCT (IOLMaster 700, Carl Zeiss Meditec AG, Dublin, CA, USA) and a surgically induced astigmatism of 0.12 D.

### 2.2. Surgical Technique

All residents were taught by the same experienced attending surgeon (KLC) and used the same surgical technique. A fixation ring and a 1.15 slit knife created the side-port incision. Healon 5^®^ (Johnson&Johnson Surgical Vision, Santa Ana, CA, USA) was injected. A 2.2 mm keratome and fixation ring created the temporal, almost clear corneal incision. A cystitome and Giannetti capsulorhexis forceps created a continuous curvilinear capsulorhexis. After hydrodissection and hydrodelineation, coaxial phacoemulsification (Whitestar Signature^®^PRO Johnson&Johnson Surgical Vision, Santa Ana, CA, USA) was performed. A divide and conquer technique was used moving to an appropriate chopping technique when skills were appropriate. Healon^®^ (Johnson&Johnson Surgical Vision, Santa Ana, CA, USA) was injected followed by injection and placement into the capsular bag of a monofocal Tecnis^®^ ZCB00 (Johnson&Johnson Surgical Vision, Santa Ana, CA, USA) [13]. The Healon^®^ was aspirated, and the incisions hydrated. Postoperatively, patients used a combination of drops (antibiotic, corticosteroid, non-steroid anti-inflammatory).

### 2.3. IOL Calculation and Optimization

For the Tecnis ZCB00, the IOLMaster 500 has a built-in, default Haigis formula with non-optimized constants. For the first 216 patients, from 11 different residents, this default Haigis formula was used for surgical planning with the IOLMaster 500, 167 patients, and with the IOLMaster 700, 49 patients.

Using the initial 128 cases, a built-in function of the IOLMaster 500 optimized a0 such that the postoperative refractive spherical equivalent (SEQ) should be closer to predicted. This built-in function specifically only optimized the a0 constant. This a0-optimized constant, with the non-optimized a1 and a2, proactively calculated IOL power for surgical planning for the subsequent 94 surgeries of seven residents.

To optimize all three Haigis constants, the postoperative SEQs for all the initial 216 patients were used. A multiple linear regression was performed to back-calculate the ELP term in the Haigis that would bring the SEQ closer to its predicted. These a0/a1/a2-optimized constants were then used proactively in surgical planning for the subsequent and final 121 surgeries for 11 residents in the study.

To evaluate the efficiency of the optimization process, biometrics from these 121 a0/a1/a2-optimized cases were input into the online Barrett Universal II (Barrett UII) https://calc.apacrs.org/barrett_universal2105/ (accessed on 30 January 2024), Kane https://www.iolformula.com/ (accessed on 30 January 2024) and Hill-RBF https://rbfcalculator.com/online/index.html (accessed on 30 January 2024) calculators. The manufacturer’s A constant of 119.3 (Kane and Hill-RBF) and lens factor (LF) of 2.09 (Barrett UII) were used [13]. The predicted SEQ that corresponded to the IOL power that was actually implanted was used for comparisons.

The ELP increment needed to reduce systematic bias in the a0/a1/a2-optimized surgeries (n = 121) was calculated using the analytical function of a thick-lens pseudophakic model [12,14]. The ELP increment was used to update manufacturer’s A constant and LF for the Tecnis ZCB00. For this a0/a1/a2-optimized group, the updated A constant and LF were input into the three online calculators to produce the predicted SEQ for the IOL power implanted.

### 2.4. Outcome Measures

Refractive prediction performance was assessed using the arithmetic mean prediction error (AME), mean absolute prediction error (MAE), median absolute prediction error (MedAE), and root mean square prediction error (RMSE) [11,15,16]. RMSE has been increasingly adopted into modern IOL formula studies as an alternative to standard deviation (SD) and was used for subgroup analysis, as recommended by Dr. Holladay [15,16]. Postoperative spherical equivalent (SEQ) prediction accuracy was further characterized by calculating the percentage of eyes within ±0.25 D, ±0.50 D, and ±1.00 D of the predicted SEQ. Best corrected visual acuity (BCVA) between groups was compared.

### 2.5. Statistical Analysis

Data were analyzed using SPSS Statistics for Windows (version 27.0, SPSS, Inc., Chicago, IL, USA). The Shapiro-Wilk test was used to check data distributions for normality. The Wilcoxon signed-rank test was used to compare differences. The bootstrap-t method with Holm correction was used to compare RMSE between groups. A *p*-value less than 0.05 was considered statistically significant. Comparisons between the clinical outcomes of the non-optimized, a0-optimized, and a0/a1/a2-optimized groups were performed. Comparisons between the a0/a1/a2-optimized group and the online calculators were performed.

## 3. Results

The average age of 431 patients was 62.73 ± 10.15 years (range 26 to 89 years). Of the 262 females and 169 males included, 47% received initial surgery on the left eye and the remaining 53% on the right eye. The average follow-up for the non-optimized, a0-optimized, and a0/a1/a2-optimized groups were 37 ± 23 days, 40 ± 24 days, and 45 ± 27 days, respectively.

The default Haigis constants from the IOLMaster 500 are listed in Table 1. The a0-optimized constant and the a0/a1/a2-optimized constants are listed in Table 1.

The mean biometrics (AL, K, ACD) for each group are listed in Table 2. The average implanted IOL powers for each group are listed in Table 2.

Table 3, Table 4 and Table 5 show the clinical effect of Haigis constant optimization used proactively for surgical planning. The absolute SEQ decreased significantly compared to the non-optimized (0.49 ± 0.57 D) to the a0-optimized group (0.32 ± 0.37 D) (*p* = 0.002) (Table 3). Similarly, the absolute SEQ of the a0/a1/a2-optimized group (0.22 ± 0.34 D) was significantly reduced from the non-optimized and a0-optimized groups (*p* < 0.001, *p* = 0.018), respectively (Table 3). Optimization increased the percentage of eyes within ± 0.50 D from 65.74% (non-optimized) to 74.47% (a0-optimized) to 95.04% (a0/a1/a2-optimized) (Table 4).

Table 5 shows the improved outcomes of optimization on the AMEs and MAEs. Using the non-optimized Haigis formula, the AME was −0.22 ± 0.54 D, and the MAE was 0.44 ± 0.38 D. Using the a0-optimized constant, significantly reduced the MAE to 0.35 ± 0.37 D (*p* = 0.009); the AME was reduced, −0.11 ± 0.50 D, but not significantly (*p* = 0.208). Compared to the non-optimized and the a0-optimized groups, the a0/a1/a2 optimization further significantly reduced the AME to 0.03 ± 0.29 D and MAE to 0.19 ± 0.22 D (*p* = 0.026 and *p* < 0.001), respectively.

Table 6 shows the distribution of preoperative and postoperative BCVA outcomes for the non-optimized, a0-optimized, and a0/a1/a2-optimized Haigis formulas. Across all three groups, a clear improvement in postoperative visual acuity was observed following optimization. The percentage of eyes achieving excellent BCVA (20/20–20/25) increased substantially, rising from 71.49% postoperatively in the non-optimized group, to 78.72% and 79.34% in the a0-optimized and a0/a1/a2-optimized groups, respectively. Simultaneously, the incidence of eyes with severely reduced postoperative BCVA (20/200 or worse) declined with each step of optimization, from 4.71% in the non-optimized group, to 2.13% in the a0-optimized group, and 0% in the a0/a1/a2-optimized group. Additionally, the proportion of eyes within the intermediate visual acuity ranges (20/30–20/70 and 20/71–20/150) decreased across all groups postoperatively, indicating a redistribution of patients into higher acuity brackets whilst minimizing poor outcomes.

Table 7 compares the MAEs of the a0/a1/a2-optimized data set with non-updated and updated A constant and LF for the online calculators (n = 121 for each). The change in ELP was −0.025 mm, and base ACD was 5.78 mm [12]. Therefore, the updated ACD is 5.755, and the updated A constant is 119.266 (A = 119.27 in online calculators, which converts to LF = 2.03 for the Barrett UII). Using the manufacturer’s LF for the Barrett UII the A constant for the Kane and Hill-RBF, the MAEs (0.25 ± 0.31 D, 0.24 ± 0.30 D, 0.23 ± 0.32 D, respectively) were slightly more than but not significantly different from the MAE of the a0/a1/a2-optimized group (0.19 ± 0.22 D) [13]. Optimization of the ELP to update the A constant and LF resulted in the MAEs for the Barrett UII, Kane, and Hill-RBF (0.21 ± 23 D, 0.21 ± 0.21 D, 0.21 ± 0.22 D) to more closely approach the MAE for the a0/a1/a2-optimized group.

Table 8 compares RMSE across all formula groups, including the online calculators with non-updated and updated A constant and LF (n = 121 for each online calculator). For the Haigis, RMSE progressively decreased with increasing levels of optimization, from 0.580 in the non-optimized group to 0.506 in the a0-optimized group, and 0.289 in the a0/a1/a2-optimized group (Table 8). The a0/a1/a2-optimized group significantly outperformed both the non-optimized (*p* < 0.001) and a0-optimized (*p* = 0.0019) groups.

The RMSE for the a0/a1/a2-optimized Haigis formula was the lowest across all formulas and was significantly lower than the Barrett UII, Kane and Hill-RBF formulas (manufacture’s A constant and LF) (*p* < 0.001, *p* < 0.001, *p* = 0.004, respectively), for the same eyes (n = 121). However, for the same eyes (n = 121), the a0/a1/a2-optimzed Haigis RMSE was not significantly lower than the ELP updated Barrett UII, Kane, or Hill-RBF formulas (*p* = 0.145, *p* = 0.158, *p* = 0.171, respectively) (Table 8).

Among the online formulas, the Kane formula had the lowest RMSE of 0.379, however it was not significantly lower than the Barrett UII or Hill-RBF formulas (*p* = 0.578 and *p* = 0.245, respectively). Using the updated A constant and LF in these formulas yielded the same results. The updated Kane formula again had the lowest RMSE of 0.294, and it was again not significantly different from either the updated Barrett UII or updated Hill-RBF formulas (*p* = 0.920 and *p* = 0.423, respectively) (Table 8).

## 4. Discussion

### 4.1. Variability in Resident Training and the Need for Evidence Based Approaches

Ophthalmology residency programs exhibit variability in training experiences and surgical skills, particularly concerning cataract and IOL implantation procedures [17,18]. While competency based curricula are standard, integrating evidence based analysis can further enhance training outcomes [5,6,19]. Previous research has established teaching milestones for cataract surgery through evidence based methods [20]. To our knowledge, our study is the first to demonstrate that evidence based optimization of manufacturer’s IOL constants and minimizing systematic bias in the ELP across multiple novice residents significantly improves refractive outcomes.

### 4.2. Understanding Biometry and IOL Calculation Formulas

A formal curriculum emphasizing the understanding of biometry and IOL calculation formulas is essential. Modern formulas such as the Hill-RBF, Emmetropia Verifying Optical (EVO) Formula, and Kane, utilize artificial intelligence, pattern recognition, and segmented axial lengths but are considered “black box” due to their unpublished methodologies [21,22]. Also, although the commonly used Barrett Universal II does not use artificial intelligence, its methodology is “black box” [21]. In contrast, the PEARL-DGS, which uses machine learning, is more transparent [23]. Mastering the instruments that measure ocular biometry, such as PCI and Scheimpflug principle biometers, is the first step in understanding IOL calculations [24]. One study showed that formalized training in biometry leads to improved refractive outcomes [25].

### 4.3. Comparing IOL Calculation Formulas in Resident Surgeries and the Importance of Constant Optimization

Evaluating the outcomes of different IOL calculation formulas is the second step in understanding resident cataract surgery results. While the Barrett UII formula is often considered highly accurate, a study at the Providence Veterans Affairs Medical Center, where residents performed the majority of surgeries, found no significant differences in MAE among Barrett UII, Holladay 2, and Hill-RBF formulas [26]. Monofocal IOLs were implanted, and constant optimization was not performed. The percentages of eyes within ±0.25 D and ±0.50 D of target refraction were also comparable across these formulas.

Importantly, in our study, optimizing just the a0 constant resulted in a MAE of 0.35 D, which is lower than the MAEs, approximately 0.40 D, reported for Barrett UII, Holladay 2, and Hill-RBF in the Providence Veterans Affairs Medical Center study [26]. Furthermore, 61.7% and 76.86% of eyes were within a postoperative SEQ of ±0.25 D with the a0-optimized and with a0/a1/a2-optimized Haigis constants, respectively, outperforming the approximately 40% reported in the aforementioned study [26].

### 4.4. Utilizing Independent Datasets for Optimization

Research emphasizes the importance of using independent datasets when comparing prediction errors between non-optimized and optimized formulas to avoid overestimating the efficacy of optimization [27]. In our study, data for each optimized group (a0 and a0/a1/a2) were compiled from independent cases of resident performed cataract surgery, adhering to this recommendation (Table 5). Additionally, a large study documented that optimizing constants for a group of surgeons exceeds any additional benefit of personalizing constants for individual surgeons, a premise supported by our findings [8].

### 4.5. Optimizing IOL Constants to Improve Refractive Outcomes

The third step for quality improvement involves evidence based optimization of IOL formula constants and used proactively to improve the predictability of the ELP and, consequently, refractive outcomes. Our study clearly demonstrates the value of optimization. Optimizing just the a0 constant significantly improved the MAE compared to the MAE of the non-optimized group (Table 5). Importantly, there was further significant improvement of the MAE in the a0/a1/a2-optimized group across multiple novice resident surgeons (Table 5). Maximum optimization resulted in 95.04% with a SEQ ± 0.50 D (Table 4).

### 4.6. Reducing Systematic Bias in ELP Prediction

It is well known that a common source of uncertainty in IOL power calculations is predicting ELP [28]. Manufacturers provide an A constant and a LF specific to the IOL to predict the ELP. Each online formula employs proprietary methods to predict ELP [21]. To account for systematic bias, evidence based updating of manufactures’ constants is now possible [12]. These updated constants can be used in “black box” formulas to have quality improvement of patient refractive outcomes.

Therefore, we compared the MAE of our maximally optimized group, a0/a1/a2, with the MAE of the online calculators, Barrett Universal II, Kane, and Hill-RBF, using both the manufacture’s constants and the updated constants (Table 7). The a0/a1/a2-optimized group had the lowest MAE. However, these differences did not reach statistical significance. Notably, when the updated A constant and LF were applied, their MAEs approached that of the a0/a1/a2-optimized Haigis formula, again with no significant differences. This is not surprising as iterative methods to optimize constants should also reduce the systematic bias for predicting the ELP.

The inclusion of RMSE as an additional outcome measure in this study provides valuable insight into the precision of refractive outcomes beyond central tendency parameters like MAE and MedAE. RMSE progressively decreased from 0.580 in the non-optimized group to 0.506 in the a0-optimized group and to 0.289 with optimization of all three Haigis constants, indicating a marked reduction in the variability and severity of outlier refractive errors (Table 8). These improvements are consistent with modern studies that emphasize RMSE as a preferred metric for evaluating formula performance in non-normally distributed error populations [11,21,29,30].

Notably, for the same eyes, the RMSE of the a0/a1/a2-optimized group was significantly lower than that of the Barrett UII, Kane, and Hill-RBF formulas when using default manufacturer constants. This reduction highlights that evidence based optimization can achieve greater consistency than even advanced “black box” methods. Furthermore, after applying the ELP updated A constant and LF to these online formulas for the same eyes, their RMSE values became statistically indistinguishable from the a0/a1/a2-optimized Haigis formula. These outcomes underscore that evidence based analysis is an important educational training tool for ophthalmology residents [6].

### 4.7. Follow-Up Duration and Refractive Stability

Our study displayed variability in patient follow-up times. The average follow-up was approximately one month post operation. Evidence suggests that for small incision clear corneal phacoemulsification, this follow-up duration is adequate for refractive stability [31].

## 5. Conclusions

Ophthalmology residency programs must optimally allocate educational time and experiences [7]. In summary, an integral component of surgical curricula should include evidence based training along with competency based numbers for cataract surgery [7]. Specifically, we demonstrated that incorporating evidence based analysis into residency education improves refractive outcomes among novice ophthalmology surgeons. We acknowledge that our results are likely influenced by the level of attending physician involvement in each case, which was determined by the proficiency of individual resident surgeons. Also, the preoperative and postoperative refraction procedures were not standardized.

## Figures and Tables

**Table 1:** Non-optimized, a0-optimized and a0/a1/a2-optimized Haigis formula constants.

Formula	a0	a1	a2
Non-optimized	+1.552	+0.400	+0.100
a0-optimized	+1.533	+0.400	+0.100
a0/a1/a2-optimized	+0.464	+0.367	+0.150

**Table 2:** Biometry measurements and implanted IOL power of formula groups.

Formula	Axial Length (mm)	Mean Keratometry (D)	Anterior Chamber Depth (mm)	Implanted IOL Power (D)
Non-optimized	23.58 ± 1.09	43.91 ± 1.69	3.22 ± 0.45	21.38 ± 3.07
a0-optimized	23.43 ± 0.97	43.98 ± 1.44	3.29 ± 0.38	23.43 ± 0.98
a0/a1/a2-optimized	23.91 ± 1.06	43.69 ± 1.57	3.27 ± 0.41	20.43 ± 2.92

Mean biometric measurements across the various patient and formula groups. Means ± SD; D = Diopter.

**Table 3:** Comparison of postoperative spherical equivalent refractions for the non-optimized and optimized Haigis formulas.

Formula	Postoperative SEQ (D)	Absolute Postoperative SEQ (D)
Non-optimized	−0.29 ± 0.70	0.49 ± 0.57
a0-optimized	−0.18 ± 0.46	0.32 ± 0.37
a0/a1/a2-optimized	−0.14 ± 0.38	0.22 ± 0.34

Comparison of mean and mean-absolute postoperative spherical equivalent refraction. Subsequent optimization of the a0 Haigis constant and the a0/a1/a2 Haigis constants displayed reduction in postoperative spherical equivalent refractions 1 month after surgery. Means ± SD; D = Diopter; SEQ = Spherical equivalent refraction.

**Table 4:** Percentage of eyes within certain prediction error ranges for the various formula groups.

Prediction Error Range	Non-Optimized Haigis (n = 216)	a0-Optimized Haigis (n = 94)	a0/a1/a2-Optimized Haigis (n = 121)
±0.25 Diopter	38.89%	58.51%	72.73%
±0.50 Diopter	65.74%	74.47%	95.04%
±1.00 Diopter	92.59%	92.55%	99.17%

Comparison of the percentage of eyes that fell within the prediction error ranges for the various formula groups.

**Table 5:** Refractive prediction errors across formula groups.

Formula	Arithmetic Mean Error (D)	Mean Absolute Error (D)	Median Absolute Error (D)
Non-optimized	−0.22 ± 0.54	0.44 ± 0.38	0.36
a0-optimized	−0.11 ± 0.50	0.35 ± 0.37	0.21
a0/a1/a2-optimized	0.03 ± 0.29	0.19 ± 0.22	0.14

Means ± SD; D = Diopter.

**Table 6:** Preoperative and Postoperative Best Corrected Visual Acuity Outcomes by Haigis Constant Optimization.

BCVA Range	Non-Optimized (Pre-Op)	Non-Optimized (Post-Op)	a0-Optimized (Pre-Op)	a0-Optimized (Post-Op)	a0/1/a2-Optimized (Pre-Op)	a0/a1/a2-Optimized (Post-Op)
20/20–20/25	8.64%	71.49%	8.60%	78.72%	6.67%	79.34%
20/30–20/70	56.79%	15.13%	55.91%	18.09%	52.50%	20.66%
20/71–20/150	7.41%	8.67%	9.68%	1.06%	19.17%	0%
20/200 or worse	27.16%	4.71%	25.81%	2.13%	21.67%	0%

Percentage of eyes within each BCVA group. Successive optimization of Haigis constants led to an increased percentage of postoperative BCVA within 20/20–20/25. Additionally, the number of eyes with poor visual outcomes (BCVA 20/71–20/150) decreased with each successive optimization step, ultimately reaching 0% in the a0/a1/a2-optimized cohort. BCVA = Best Corrected Visual Acuity.

**Table 7:** Comparison of MAE for the a0/a1/a2-optimized Haigis formula vs. three online formulas (n = 121) with manufacture’s A constant and LF and with ELP updated A constant and LF.

Formula	MAE (D)	*p*-Value
a0/a1/a2-optimized Haigis	0.19 ± 0.22	
A = 119.30, LF = 2.09		
Barrett UII	0.25 ± 0.31	0.096
Kane	0.24 ± 0.30	0.069
Hill-RBF	0.23 ± 0.32	0.144
A = 119.27, LF = 2.03		
Barrett UII	0.21 ± 0.23	0.268
Kane	0.21 ± 0.21	0.351
Hill-RBF	0.21 ± 0.22	0.499

Comparison of MAE for the a0/a1/a2-optimized Haigis formula vs. Barrett UII, Kane and Hill-RBF formulas. The manufacturer’s A constant = 119.30 was used for the Hill-RBF and Kane formulas. The manufacturer’s LF = 2.09 was used for Barrett UII. Updated ELP updated the A constant (119.27) (Hill-RBF and Kane) and the LF (2.03) (Barrett UII). Means ± SD; D = Diopter; LF = lens factor; MAE = mean absolute error.

**Table 8:** Comparison of RMSE across all formulas.

Formula	RMSE (D)
Non-optimized Haigis	0.580
a0-optimized Haigis	0.506
a0/a1/a2-optimized Haigis	0.289
A = 119.30, LF = 2.09 (n = 121)	
Barrett UII	0.392
Kane	0.379
Hill-RBF	0.435
A = 119.27, LF = 2.03 (n = 121)	
Barrett UII	0.311
Kane	0.294
Hill-RBF	0.302

Comparison of RMSE for the non-optimized, a0-optimized and a0/a1/a2-optimized Haigis formula and Barrett UII, Kane, and Hill-RBF formulas. The manufacturer’s A constant = 119.30 was used for the Hill-RBF and Kane formulas. The manufacturer’s LF = 2.09 was used for Barrett UII. Updated ELP updated the A constant (119.27) (Hill-RBF and Kane) and the LF (2.03) (Barrett UII). D = Diopter; RMSE = Root mean square prediction error

## Data Availability

The data that support the findings of this study are available from the corresponding author upon reasonable request.

## Abbreviations

The following abbreviations are used in this manuscript:

IOL	Intraocular lens
ELP	Effective lens position
PCI	Partial coherence interferometry
SEQ	Spherical equivalent refraction
Barrett UII	Barrett Universal II
LF	Lens factor
AME	Arithmetic mean error
MAE	Mean absolute error
MedAE	Median absolute error
RMSE	Root mean square prediction error
BCVA	Best corrected visual acuity
AL	Axial length
K	Keratometry
ACD	Anterior chamber depth
